# Supplementary material for: National COVID-19 lockdown and trends in help-seeking for violence against children in Zimbabwe: an interrupted time-series analysis
Source: BMC Public Health. 2022 Nov 18;22:2116. doi: 10.1186/s12889-022-14425-w (PMC9673211; doi:10.1186/s12889-022-14425-w)
Supplement: Supplementary file 1 — Additional file 1. STROBE Statement—checklist of items that should be included in reports of observational studies [file 12889_2022_14425_MOESM1_ESM.docx]

**Additional file 1. STROBE Statement—checklist of items that should be included in reports of observational studies**

|  | Item No. | Recommendation | Page  No. | Relevant text from manuscript |
| --- | --- | --- | --- | --- |
| **Title and abstract** | 1 | (*a*) Indicate the study’s design with a commonly used term in the title or the abstract | 2 | ‘We used an interrupted time series design’ |
|  |  | (*b*) Provide in the abstract an informative and balanced summary of what was done and what was found | 2 | **‘Methods**  We used an interrupted time series design to analyse the proportion of violence related calls (17,913 calls out of 57,050) to Childline Zimbabwe’s national child helpline between 2017 to 2021. We applied autoregressive integrated moving average regression (ARIMA) models to test possible changes in call trends before and after the March 2020 lockdown and forecasted how many calls would have been received in the absence of lockdown. In addition, we examined call characteristics before and after lockdown descriptively.  **Results**  The proportion of violence related calls decreased in the 90 days after the lockdown and subsequently returned to pre-COVID-19 levels. We estimate that 10.3% (95% confidence interval [CI] 6.0-14.6%) more violence related calls would have occurred in this period had there not been a lockdown. Violence was increasingly reported as occurring in children’s households, with fewer reports from children and formal child protection actors’ |
| Introduction | | | |  |
| Background/rationale | 2 | Explain the scientific background and rationale for the investigation being reported | 4-5 | ‘Mobility restrictions, such as ‘lockdowns’, were invaluable in preventing the spread of infection from the coronavirus disease of 2019 (COVID-19). Lockdowns may also have had unintended consequences in increasing the risk of violence against children. Households experienced stress, financial loss and other negative consequences as a result of lockdown measures, which increased children’s risk of violence [1–3]. Greater time spent in households similarly may have increased victimisation in cases where abuse was ongoing and where household members themselves were experiencing elevated levels of stress [4]. Several studies documented short-term spikes in help-seeking for domestic violence against women and children in the weeks after initiation of stay-at-home or shelter-in-place orders in the United States [2,5,6], Germany [7], Peru [8], and Argentina [9]. Likewise, caregivers with a lack of social support had nearly doubled the odds of physical and emotional violence against children in the first two weeks after social distancing measures were instituted in the United States [1].    Whilst COVID-19-related lockdowns may create or exacerbate conditions that increase violence against children, they also close traditional avenues for help-seeking [11]. Healthcare and social service referrals were curtailed during lockdown periods, leading to lower detection of violence [12,13]. Remote forms of reporting violence, therefore, become increasingly important as a mechanism for help-seeking, particularly as lockdown measures extend over multiple months. Child helplines are usually toll-free numbers that are widely available in most countries [11], and many child helplines continue to operate as essential services during COVID-19 lockdowns. Although child helplines experienced reductions in capacity to answer calls like other services providers, they remained one of the few avenues for continued reporting of violence against children [14].  It is important to isolate how and when national COVID-19 containment policies may have impacted children’s ability to seek help, especially in contexts like sub-Saharan Africa where violence reporting and formal service provision were limited prior to the pandemic. A former multi-country study found that a range of under 1% to 25% of children disclose violence to a formal child protection actor in the region [15]. During the COVID-19 pandemic, social service providers in Kenya, Uganda, Nigeria, and South Africa stated that violence prevention and response decreased even further due to government restrictions and a lack of prioritisation of violence programmes [16]. In particular, past studies identified educational personnel as the most prominent formal actor for child disclosure of violence in sub-Saharan Africa [15,17]. The COVID-19 lockdown closed this traditional avenue for help-seeking in countries like Zimbabwe. A growing number of prevalence studies identified increases in violence against women and children (for example, in Uganda and South Africa [18,19]), but only one other study to our knowledge examined violence help-seeking. Decker et al. [20] prospectively found that women and girls in Nairobi, Kenya increased help-seeking for intimate partner violence and sexual violence during the pandemic. Our study expands the evidence for sub-Saharan Africa as a region and more broadly, contributes to a greater understanding on how COVID-19 policies affected help-seeking patterns in resource constrained settings. We apply the most rigorous methodology to date for studying how COVID-19 policy changes influenced violence help-seeking trends in sub-Saharan Africa, isolating the impact of a national lockdown and temporal trends for violence help-seeking’ |
| Objectives | 3 | State specific objectives, including any prespecified hypotheses | 4,5 | p.4: ‘Lockdowns may have unintended consequences, however, including increasing the risk of violence against children’  p.4: ‘Whilst COVID-19-related lockdowns may create or exacerbate conditions that increase violence against children, they also close traditional avenues for help-seeking [11].’  p.5: ‘Our specific objectives were to: (a) test hypotheses about how the implementation of lockdown measures might change violence call patterns to Childline Zimbabwe, (b) estimate how many calls for violence would have been received in the absence of the national lockdown and (c) describe the characteristics of calls before and after the national COVID-19 lockdown’ |
| Methods | | | |  |
| Study design | 4 | Present key elements of study design early in the paper | 6 | ‘We applied a quasi-experimental, interrupted time series design supplemented by descriptive statistics to understand changes in help-seeking for violence against children before and after the government of Zimbabwe’s implementation of a national COVID-19 lockdown’ |
| Setting | 5 | Describe the setting, locations, and relevant dates, including periods of recruitment, exposure, follow-up, and data collection | 6-8 | ‘**Zimbabwean context**  Zimbabwe, bordering South Africa to the north, has a long history of economic and social interchange with its southern neighbour and shares many similarities [21]. Zimbabwe is also representative of other sub-Saharan African countries in its characteristics. In recent years, it was approximately average in its economic growth as compared to other African nations, occupying the lower quartile of gross domestic products (GDP) and on par with Mauritania and Sierra Leone. Approximately a third of the country was rural and slightly higher than 60% of the country was employed in the informal sector, which is roughly the average across the continent [22]. The levels of violence and service provision structures are likewise comparable regionally. Between 26% and 27% of boys and girls experienced violence before the age of 18 [23]. These percentages are similar to Uganda and Nigeria, which respectively have an estimated 28% and 30% prevalence of violence against children [24]. While Zimbabwe has ratified extensive child rights and protection legislation, a barrier to full implementation is adequate and stable government financing [25]. Zimbabwe, like many other similar countries regionally and globally, underfunds social protection as a percentage of its national budget and has yet to realise a fully integrated and comprehensive national child protection system [26,27].  **National COVID-19 lockdown**  On 30^th^ March 2020, the government of Zimbabwe instituted a strict national lockdown to hinder community spread of COVID-19. The associated public health order prohibited individuals from leaving their households except for accessing essential services within a radius of five kilometres from their homes. Schools and other places of gathering, however, were closed [28]. A substantial number of police and military were deployed throughout the country to enforce infractions of COVID-19 regulations. It was estimated that between March and July 2020, over 100,000 were arrested, which was more than the cumulative number of COVID-19 cases in the country [29,30]. Public health orders, issued fortnightly, extended lockdown measures until 17^th^ May 2020 when individuals were permitted to leave their homes if masked [28,31]. Mobility restrictions were again tightened on 22^nd^ July 2020 when an evening curfew was implemented [32]. The Ministry of Primary and Secondary Education instituted a phased reopening of schools between September and November 2020, with a month of complete opening followed by reinstitution of strict lockdown measures in January 2021 that extended until the end of the study period in February 2021 [33,34]. This study uses the national lockdown on 30^th^ March 2020 as the intervention of interest, which is the clearest time point of policy enactment.  **Sample**  Childline is the only nationwide helpline service in Zimbabwe dedicated to children. It operates a 24-hour, toll-free number from which children or concerned individuals can call and obtain information or services [35]. All calls are automatically logged by date and provided with a unique identification number. We obtained anonymised call data for 48 months from 20^th^ February 2017 until 20^th^ February 2021. Calls originated from service providers, caregivers or other concerned adults and involved cases against children committed by adults and violence committed by or against peers’ |
| Participants | 6 | (*a*) *Cohort study*—Give the eligibility criteria, and the sources and methods of selection of participants. Describe methods of follow-up  *Case-control study*—Give the eligibility criteria, and the sources and methods of case ascertainment and control selection. Give the rationale for the choice of cases and controls  *Cross-sectional study*—Give the eligibility criteria, and the sources and methods of selection of participants | 7-8 | ‘We obtained anonymised call data for 48 months from 20^th^ February 2017 until 20^th^ February 2021. Calls originated from service providers, caregivers or other concerned adults and involved cases against children committed by adults and violence committed by or against peers’ |
|  |  | (*b*) *Cohort study*—For matched studies, give matching criteria and number of exposed and unexposed  *Case-control study*—For matched studies, give matching criteria and the number of controls per case |  |  |
| Variables | 7 | Clearly define all outcomes, exposures, predictors, potential confounders, and effect modifiers. Give diagnostic criteria, if applicable | 7,8 | p.7: ‘This study uses the national lockdown on 30^th^ March 2020 as the intervention of interest, which is the clearest time point of policy enactment’  p.8: ‘**Outcome variables**  Call operators designated if callers were seeking help for one or multiple forms of physical, emotional or sexual violence against children, bullying or cases of domestic violence with children in the household. We indicated a call as related to violence if it was categorised as one of these five forms of violence. Childline Zimbabwe defines physical violence as force used against a child with the intention to injure. Emotional violence is defined as psychologically harmful acts towards a child. Sexual violence includes sexual harassment, sexual assault (except for rape and forced sex), rape, sexual coercion and exploitation, exposure to pornography, and verbal sexual harassment. Bullying is perpetrated by children against other children and entails the use of power and aggression to cause harm or distress. Domestic violence are acts of any type of abuse within a child’s household (personal communication, Beaulah Nengomasha, 2021)’ |
| Data sources/ measurement | 8* | For each variable of interest, give sources of data and details of methods of assessment (measurement). Describe comparability of assessment methods if there is more than one group | 7-8 | ‘All calls are automatically logged by date and provided with a unique identification number. We obtained routine, anonymised call data for 48 months from 20^th^ February 2017 until 20^th^ February 2021. Calls originated from service providers, caregivers or other concerned adults and involved cases against children committed by adults and violence committed by or against peers’ |
| Bias | 9 | Describe any efforts to address potential sources of bias | 8-9 | p.8-9: ‘ARIMA models are useful in interrupted time series analyses as they can accommodate seasonality and autocorrelation better than traditional segmented regression [36]. Childline Zimbabwe closed some of its call centres after lockdown measures were instituted, so we specifically modelled the proportion of violence related calls out of the total number of daily calls to account for reductions in child helpline capacity over the course of the study’  p.9:’We created three ARIMA models to test different temporal patterns of help-seeking…’  P.9:‘We followed a set protocol for automated selection of each ARIMA model [36]. The stationarity of variance was assessed quantitatively by applying Goldfeld-Quandt tests for heteroscedasticity (*p-*value < 0.10). Autocorrelation was examined visually in correlograms and partial correlograms. We allowed the algorithm to select the best fitting ARIMA model with the smallest Akaike Information Criterion (AIC) [37]. Visual checks were conducted to determine if residual autocorrelation was present after model fitting’ |
| Study size | 10 | Explain how the study size was arrived at | 7 | ‘We obtained anonymised call data for 48 months from 20^th^ February 2017 until 20^th^ February 2021’ |

Continued on next page

| Quantitative variables | 11 | Explain how quantitative variables were handled in the analyses. If applicable, describe which groupings were chosen and why | 8-9 | P.8:‘We indicated a call as related to violence if it was categorised as one of these five forms of violence’  P.8-9:‘Childline Zimbabwe closed some of its call centres after lockdown measures were instituted, so we specifically modelled the proportion of violence related calls out of the total number of daily calls to account for reductions in child helpline capacity over the course of the study’  P.8:‘[The number of] calls would have been received for physical, emotional and sexual violence, bullying and domestic violence for girls and boys in the absence of COVID-19 policy enactment’ |
| --- | --- | --- | --- | --- |
| Statistical methods | 12 | (*a*) Describe all statistical methods, including those used to control for confounding | 8-9 | p.8-9: ‘Our analysis consisted of two main components. We created autoregressive integrated moving average regression (ARIMA) models to quantify potential changes in help-seeking following the national lockdown in March 2020 and to estimate the counterfactual of how many calls would have been received for physical, emotional and sexual violence, bullying and domestic violence for girls and boys in the absence of COVID-19 policy enactment. ARIMA models are useful in interrupted time series analyses as they can accommodate seasonality and autocorrelation better than traditional segmented regression [36]. Childline Zimbabwe closed some of its call centres after lockdown measures were instituted, so we specifically modelled the proportion of violence related calls out of the total number of daily calls to account for reductions in child helpline capacity over the course of the study. We further described the characteristics of calls and callers before and after the national COVID-19 lockdown to examine data descriptively’  p.9: ‘We followed a set protocol for automated selection of each ARIMA model [36]. The stationarity of variance was assessed quantitatively by applying Goldfeld-Quandt tests for heteroscedasticity (*p-*value < 0.10). Autocorrelation was examined visually in correlograms and partial correlograms. We allowed the algorithm to select the best fitting ARIMA model with the smallest Akaike Information Criterion (AIC) [37]. Visual checks were conducted to determine if residual autocorrelation was present after model fitting. The data was analysed using R statistical software, version 4.0.3 [38]’ |
|  |  | (*b*) Describe any methods used to examine subgroups and interactions | NA | - |
|  |  | (*c*) Explain how missing data were addressed | NA | - |
|  |  | (*d*) *Cohort study*—If applicable, explain how loss to follow-up was addressed  *Case-control study*—If applicable, explain how matching of cases and controls was addressed  *Cross-sectional study*—If applicable, describe analytical methods taking account of sampling strategy | NA | - |
|  |  | (*e*) Describe any sensitivity analyses | 9 | ‘We created three ARIMA models to test different temporal patterns of help-seeking and subsequently, forecasted the proportion of calls that would have been received had the national lockdown not occurred in the specific time period. These models were: (a) the proportion of violence calls in the time period before and after national lockdown to suggest an overall change (*lockdown model*), (b) the proportion of violence calls in the 90 days after national lockdown to denote a short-term but temporary effect (*90 day model*) and (c) the average monthly proportion of calls related to violence after national lockdown to identify sustained effects (*monthly average model*). All call data prior to March 2020 was utilised in the modelling and compared to 11 months, 90 days and monthly averages of calls after the national lockdown’ |
| Results | | | | |
| Participants | 13* | (a) Report numbers of individuals at each stage of study—eg numbers potentially eligible, examined for eligibility, confirmed eligible, included in the study, completing follow-up, and analysed | 10 | ‘A total of 57,050 unique calls were received during the period of data collection of which 17,913 were related to one or multiple forms of violence against children’ |
|  |  | (b) Give reasons for non-participation at each stage | NA | - |
|  |  | (c) Consider use of a flow diagram | NA | - |
| Descriptive data | 14* | (a) Give characteristics of study participants (eg demographic, clinical, social) and information on exposures and potential confounders | Additional file 3 | Children’s gender (%), Children’s age (mean, SD), caller’s identity (%), location of violence (%), perpetrator’s gender (%), perpetrator’s relationship to victim (%) |
|  |  | (b) Indicate number of participants with missing data for each variable of interest | Additional file 3 | Unknown categories |
|  |  | (c) *Cohort study*—Summarise follow-up time (eg, average and total amount) |  |  |
| Outcome data | 15* | *Cohort study*—Report numbers of outcome events or summary measures over time |  |  |
|  |  | *Case-control study—*Report numbers in each exposure category, or summary measures of exposure |  |  |
|  |  | *Cross-sectional study—*Report numbers of outcome events or summary measures | 10 | ‘Physical violence was most commonly reported in nearly half of the calls related to violence, followed by sexual and emotional violence in approximately 34.89% and 17.72% of calls. 67.81% of reported cases were perpetrated against girls. Children, themselves, most commonly reported violence committed against them. Neighbours, mothers and community members were the next most common reporters. When information on the location of violence incidents or perpetrators was indicated, the highest percentage of cases were reported as occurring within the child’s household (43.09%) and as being committed by adult male relatives, especially fathers and uncles [see Additional File 2]’ |
| Main results | 16 | (*a*) Give unadjusted estimates and, if applicable, confounder-adjusted estimates and their precision (eg, 95% confidence interval). Make clear which confounders were adjusted for and why they were included | 11, Table 1 | ‘**Predicted changes in call trends**  Our estimates suggest that the national COVID-19 lockdown in Zimbabwe led to a slight long-term decrease in violence related calls over the subsequent 11 months but no reduction in the average number of violence related calls per month. An examination of the 90 days following the lockdown, however, indicated that Childline Zimbabwe experienced a short-term decrease in violence related calls. We expect that Childline Zimbabwe would have received approximately 10% more violence related calls in the absence of the national lockdown between April to June 2020 (Table 1)’ |
|  |  | (*b*) Report category boundaries when continuous variables were categorized | NA | - |
|  |  | (*c*) If relevant, consider translating estimates of relative risk into absolute risk for a meaningful time period | NA | - |

Continued on next page

| Other analyses | 17 | Report other analyses done—eg analyses of subgroups and interactions, and sensitivity analyses | 12-13, 26-31 (Table 2) | **Data description for the 90 days before and after national lockdown**  Violence related calls decreased in the 90 days after the national COVID-19 lockdown (before: 1527, after: 493) (Table 2). A large dip additionally occurred in June 2020, which was unlike seasonal or past trends. In June, Childline received 0 calls related to violence and 2718 calls related to other issues. This event did not correspond with past call trends and lingered after accounting for any reductions in call operator capacity.  Violence reporting by children’s aunts, mothers and community members increased in the 90 days after the lockdown, whereas self-reporting of violence by children dropped by 4%. Violence incidents also were reported as occurring more often in children’s households and less frequently in schools. The difference in perpetrator identities may reflect this change in violence location. A higher percentage of perpetrators were reported to be members of children’s families, particularly stepparents. In contrast, help-seeking for violence committed by teachers decreased by six-fold, which likely reflects school closures. The potential shift in violence to the home may be further reflected in violence patterns. Bullying and sexual violence reportedly decreased but help-seeking for physical and domestic violence increased by approximately 3% (Table 2)’ |
| --- | --- | --- | --- | --- |
| Discussion | | | | |
| Key results | 18 | Summarise key results with reference to study objectives | 13-14 | ‘**Main findings of the present study**  Our study is one of the most rigorous studies to date on how national COVID-19 lockdown affected help-seeking for violence against children outside of a high-income country and in sub-Saharan Africa. Childline Zimbabwe received the highest number of calls on record after the national COVID-19 lockdown in March 2020. Violence related calls, however, slightly decreased in the 11 months after March 2020. The largest decrease in violence related calls occurred in the 90 days after the lockdown and diverged from the expected trend, particularly in the month of June 2020. We accounted for seasonal variation, trends in the data and changes in call operator capacity, and this steep drop in calls remained. We predicted that Childline would have received 10% more violence related calls between April to June 2020 had there not been a national COVID-19 lockdown in Zimbabwe. We found slight shifts in the characteristics of calls in the three-month time period after the national COVID-19 lockdown. Callers increasingly reported incidents of violence within children’s households and less in the public sphere. Whilst restrictions led to decreases in calls related to bullying and violence committed by non-relatives, a higher percentage of callers reported physical and emotional violence committed by adult male family members’ |
| Limitations | 19 | Discuss limitations of the study, taking into account sources of potential bias or imprecision. Discuss both direction and magnitude of any potential bias | 17-18 | ‘**Strengths and limitations**  Our analysis was based upon a large national dataset that provided one of the most consistent sources of information on violence help-seeking in Zimbabwe before and after the lockdown. We had access to several years of data to identify call trends. We applied a rigorous methodology to identify changes in call trends, which accounted for seasonality, secular trends and changes in operator capacity. Our data indicate a clear short-term decrease in the proportion of calls related to violence, which is unique from past time periods. We used the first and most strict national COVID-19 lockdown as a clear timepoint for our analysis. The steep decline in violence calls after March 2020 was not replicated after subsequent lockdowns. For instance, the government of Zimbabwe reinstituted lockdown measures on 22^nd^ July 2020, and schools were partially closed in mid-December, with a hard lockdown in January that extended until the end of the study period in February 2021 [32–34]. No other time period showed a marked decrease in calls related to violence. Nevertheless, similar to other time series analyses, we cannot exclude the possibility that events besides the national COVID-19 lockdown were associated with the steep drop in calls in June 2020. Second, we estimated potential changes in call trends after the initial and most strict national lockdown. The subsequent policies implemented after June 2020 resulted in lockdowns with varying levels of leniency. It is beyond the scope of this study to estimate how these changes in implementation may have influenced call patterns. Third, slight residual autocorrelation may still have been present after model fitting for the *lockdown* and *monthly average models*. Automation of the model selection process, however, identified the best fitting ARIMA models for the data without overfitting, which produces results that are generalizable beyond this dataset. Fourth, our results are not indicative of long-term changes and should only be considered valid for the duration of the study period. Fifth, estimates based upon call data should not be interpreted as violence prevalence, as most cases of violence against children are unreported. A recent analysis of nationally representative data on 13-to-17 year olds from six low- and middle-income countries indicated that a range of 23.05 to 54.13% of young people reported violence to an informal channel, and only 0.11 to 25.3% of young people reported violence to formal channels like child helplines [15]. In Zimbabwe, it is estimated that 11.9% of girls between the ages of 13 and 17 sought help for an incident of sexual violence and of those girls, 3.3% from a service provider or authority figure [23]. Finally, all information related to the characteristics of violence calls or identity of callers in the time period before and after the lockdown is purely descriptive. We had sufficient information to isolate temporal changes in call trends and estimate the proportion of violence related calls that would be likely in the absence of the COVID-19 national lockdown in Zimbabwe. The descriptive analyses are meant to provide potential nuance but should be treated as purely speculative about possible changes in help-seeking patterns’ |
| Interpretation | 20 | Give a cautious overall interpretation of results considering objectives, limitations, multiplicity of analyses, results from similar studies, and other relevant evidence | 14-15, 15-17 | p.14-15: ‘**Comparison with other studies**  ‘The findings of this study complement previous research on violence help-seeking after COVID-19 mobility restrictions. A handful of studies found that violence help-seeking from formal channels temporarily decreased in the United States [39,40], which contrast with other studies from North America, Europe and Latin America that found increases in help-seeking [2,5–9]. As a regional counterpoint, a prospective study from Kenya that surveyed women and girls before and during COVID-19 saw an increase in help-seeking for intimate partner violence by 11.1% and sexual violence by 4.6% [20]. Violence help-seeking seems to vary tremendously and be nationally and regionally specific. In addition, different COVID-19 policies may trigger unique patterns of help-seeking. In one of the few studies that rigorously examined child helpline data, Ortiz et al. [40] found that calls increased after declaration of national health emergency but decreased after school closures in the United States. Likewise, qualitative interviews that accompanied the Kenyan study indicated that curtailed movement from curfews were a major driver of violence [20].  It is not immediately clear why the proportion of calls to Childline Zimbabwe about violence declined in the period after the national COVID-19 lockdown. Call operators indicated that individuals were increasingly asking for information about the transmission of COVID-19 and had poverty-related concerns. Other pressing concerns—such as contracting the virus and securing food, water and shelter—could have superseded reporting of violence in this time period. Increased information gathering would be expected as a response to the unknowns at the beginning of a lockdown period. Zimbabwe, furthermore, was in the midst of its first recession in over a decade. Hyperinflation, unemployment and food insecurity skyrocketed in 2019. By 2020, Zimbabwe experienced an eight percent drop in GDP, and in the first half of 2020, prices were increasing by double digits each month. Estimates for the number of Zimbabweans living in extreme poverty ballooned to 49% of the population [30]. Another possibility is that household members and children may have had less privacy to report issues and particularly, seek help for sensitive issues, such as violence. The public health declaration instituted stay-at-home orders, which may have led to a higher number of people in households for longer time periods [28]. In Zimbabwe, extended families live together, particularly in high-density urban areas and rural regions [42]. When coupled with widespread job loss and school closure, the national lockdown would likely lead to greater overlap between multiple household members. The decrease in the proportion of children who self-reported violence after the national COVID-19 lockdown may reflect children’s inability to report confidentially during this time period’  pp.15-17: ‘**Implications and explanation of findings**  Our analysis indicated that the proportion of violence calls dropped in the three-month period after a national lockdown in Zimbabwe. An explanation of this call pattern could be a real decrease in the prevalence of violence against children; however, the global pandemic and national lockdown in Zimbabwe were likely perceived as stressful events that would be unlikely to reduce overall violence prevalence. Evidence of widespread loss of livelihoods, increase in mental health conditions and the rise of substance abuse in Zimbabwe support the hypothesis that the pandemic was stressful on the population level, and these are all factors associated with violence against children [41,43–47]. Rather, it is likely that help-seeking for violence decreased in this time period. The patterning of help-seeking suggests that violence prevention, reporting and response services need to be provided to children, particularly in the initial months after national lockdowns. This recommendation is likely even more important when governments enact policies that heavily restrict movement outside of households, as was the case after the lockdown in Zimbabwe. Government and child protection actors should invest in a composite approach that strengthens remote avenues for seeking help when children are confined to their households, ensures COVID-19 safe continuation of services that is geographically distributed across communities to provide equal access, establishes new community-based sites in non-traditional venues when necessary to supplement existing services, and allows for limited in-person home visitation for previously known cases of violence.  The potential shift in the profile of violence perpetrator to adult male relatives and reduction in reporting from key child protection actors outside of the home, such as teachers, emphasises the likely need for uninterrupted remote and web-based services for violence help-seeking. Globally, an estimated 1.8 billion children live in countries where COVID-19 has disrupted violence prevention and response services [14]. Child helplines are an important reporting and response service that can continue to operate after pandemic public health policies are instituted. Continued and sufficient support should be provided to these agencies after national lockdowns and in anticipation of future shocks, partnerships should be established to increase their preparedness. Our data further suggest that adult female family members and neighbours submitted a higher proportion of violence reports in the months after the national COVID-19 lockdown. Children themselves may be unable to report cases of violence, especially when committed by family members. Child protection campaigns that strengthen recognition of violence among older female family members and encourage adult women and neighbours to report suspected violence cases would aid in strengthening help-seeking reporting after lockdowns are instituted’ |
| Generalisability | 21 | Discuss the generalisability (external validity) of the study results | 17,18 | p.17: ‘Our analysis was based upon a large national dataset that provided one of the most consistent sources of information on violence help-seeking in Zimbabwe before and after the lockdown’  p.18: ‘Third, slight residual autocorrelation may still have been present after model fitting for the *lockdown* and *monthly average models*. Automation of the model selection process, however, identified the best fitting ARIMA models for the data without overfitting, which produces results that are generalizable beyond this dataset’ |
| Other information | |  | | |
| Funding | 22 | Give the source of funding and the role of the funders for the present study and, if applicable, for the original study on which the present article is based | 20 | ‘This work was supported by Stichting Benevolentia [GR-073089]. The funder had no role in the design of the study; collection, analysis, and interpretation of the data; or the writing of the manuscript’ |
|  |  |  |  |  |

*Give information separately for cases and controls in case-control studies and, if applicable, for exposed and unexposed groups in cohort and cross-sectional studies.

**Note:** An Explanation and Elaboration article discusses each checklist item and gives methodological background and published examples of transparent reporting. The STROBE checklist is best used in conjunction with this article (freely available on the Web sites of PLoS Medicine at http://www.plosmedicine.org/, Annals of Internal Medicine at http://www.annals.org/, and Epidemiology at http://www.epidem.com/). Information on the STROBE Initiative is available at [www.strobe-statement.org](http://www.strobe-statement.org).

**Article title:**

National COVID-19 lockdown and trends in help-seeking for violence against children in Zimbabwe: an interrupted time-series analysis

**Journal name:**

BMC Public Health

**Author names:**

Ilan Cerna-Turoff^1,3*^, Robert Nyakuwa^2^, Ellen Turner^3^, Charles Muchemwa Nherera^4^, Tendai Nhenga-Chakarisa^5^, Karen Devries^3^

**Affiliations:**

^1^ Department of Environmental Health Sciences, Mailman School of Public Health, Columbia University, New York, United States of America

^2^ Q Partnership, Harare, Zimbabwe

^3^ Faculty of Public Health and Policy, Department of Global Health and Development, London School of Hygiene and Tropical Medicine, London, United Kingdom

^4^ Department of Art Design and Technology Education, University of Zimbabwe, Harare, Zimbabwe

^5^ Child Rights Research Centre, Africa University, Harare, Zimbabwe

**Corresponding author:**

[it2208@caa.columbia.edu](mailto:it2208@caa.columbia.edu)
