# Supplementary material for: National COVID-19 lockdown and trends in help-seeking for violence against children in Zimbabwe: an interrupted time-series analysis
Source: BMC Public Health. 2022 Nov 18;22:2116. doi: 10.1186/s12889-022-14425-w (PMC9673211; doi:10.1186/s12889-022-14425-w)
Supplement: Supplementary file 2 — Additional file 2. Characteristics of violence related calls in the sample [file 12889_2022_14425_MOESM2_ESM.docx]

**Additional file 2. Characteristics of violence related calls in the sample**

| Characteristic |  | Total calls |
| --- | --- | --- |
|  |  |  |
| Child’s gender |  |  |
|  | Boys | 5013 (27.99%) |
|  | Girls | 12 147 (67.81%) |
|  | Unknown | 753 (4.20%) |
| Child’s age –  Mean (SD) |  |  |
|  |  | 12.12 (4.43) |
| Violence type |  |  |
|  | Bullying | 423 (2.36%) |
|  | Domestic | 973 (5.43%) |
|  | Emotional | 3174 (17.72%) |
|  | Physical | 8998 (50.23%) |
|  | Sexual | 6250 (34.89%) |
| Caller’s identity |  |  |
|  | Aunt | 708 (3.95%) |
|  | Brother | 255 (1.42%) |
|  | Caregiver/guardian | 52 (0.29%) |
|  | Community member | 1674 (9.35%) |
|  | Cousin | 202 (1.13%) |
|  | Doctor/health worker | 77 (0.43%) |
|  | Employer/colleague | 16 (0.09%) |
|  | Father | 656 (3.66%) |
|  | Friend | 741 (4.14%) |
|  | Grandparents | 217 (1.21%) |
|  | Mother | 1859 (10.38%) |
|  | Neighbour | 3173 (17.71%) |
|  | Nephew | 13 (0.07%) |
|  | Niece | 25 (0.14%) |
|  | Police | 73 (0.41%) |
|  | Self | 6200 (34.61%) |
|  | Sister | 345 (1.93%) |
|  | Social worker | 53 (0.30%) |
|  | Spouse/partner | 22 (0.12%) |
|  | Stepparent | 55 (0.31%) |
|  | Stranger | 162 (0.90%) |
|  | Student | 51 (0.28%) |
|  | Teacher | 269 (1.50%) |
|  | Uncle | 503 (2.81%) |
|  | Other | 207 (1.16%) |
|  | Unknown | 305 (1.70%) |
| Location of violence |  |  |
|  | Child’s household | 7719 (43.09%) |
|  | Child’s household and neighbourhood | 156 (0.87%) |
|  | Child’s household and school | 22 (0.12%) |
|  | Medical facility | 1 (0.01%) |
|  | Neighbourhood | 795 (4.44%) |
|  | Neighbourhood and school | 6 (0.03%) |
|  | Perpetrator’s household | 967 (5.40%) |
|  | Perpetrator’s household and school | 4 (0.02%) |
|  | Police station/prison | 220 (1.23%) |
|  | Public space | 237 (1.32%) |
|  | Religious institution | 5 (0.03%) |
|  | School | 422 (2.36%) |
|  | Women’s shelter | 101 (0.56%) |
|  | Other | 102 (0.57%) |
|  | Unknown | 7156 (39.95%) |
| Perpetrator gender |  |  |
|  | Female | 4534 (25.31%) |
|  | Male | 9006 (50.28%) |
|  | Unknown | 4373 (24.41%) |
| Perpetrator relation to victim |  |  |
|  | Aunt | 844 (4.71%) |
|  | Brother | 293 (1.64%) |
|  | Caregiver/guardian | 182 (1.02%) |
|  | Community member | 826 (4.61%) |
|  | Cousin | 237 (1.32%) |
|  | Doctor/health worker | 7 (0.04%) |
|  | Employer/colleague | 96 (0.54%) |
|  | Father | 2484 (13.87%) |
|  | Friend | 168 (0.94%) |
|  | Grandparents | 953 (5.32%) |
|  | Mother | 1010 (5.32%) |
|  | Neighbour | 1068 (5.96%) |
|  | Nephew | 38 (0.21%) |
|  | Niece | 12 (0.07%) |
|  | Police | 25 (0.14%) |
|  | Self | 31 (0.17%) |
|  | Sister | 95 (0.53%) |
|  | Social worker | 3 (0.02%) |
|  | Spouse/partner | 524 (2.93%) |
|  | Stepparent | 1707 (9.53%) |
|  | Stranger | 495 (2.76%) |
|  | Student | 106 (0.59%) |
|  | Teacher | 430 (2.40%) |
|  | Uncle | 1388 (7.75%) |
|  | Other | 954 (5.33%) |
|  | Unknown | 3937 (21.98%) |

^a^ SD = standard deviation. Percentages calculated from calls related specifically to violence and rounded to two decimal places. The total percentage of violence types is greater than 100% due to reporting of multiple victimization of children. The date range for calls was from 20^th^ February 2017 until 20^th^ February 2021. The ‘Self’ category indicates self-reporting of violence committed against another child. The ‘Student’ category refers to a pupil of the child’s school.

**Article title:**

National COVID-19 lockdown and trends in help-seeking for violence against children in Zimbabwe: an interrupted time-series analysis

**Journal name:**

BMC Public Health

**Author names:**

Ilan Cerna-Turoff^1,3*^, Robert Nyakuwa^2^, Ellen Turner^3^, Charles Muchemwa Nherera^4^, Tendai Nhenga-Chakarisa^5^, Karen Devries^3^

**Affiliations:**

^1^ Department of Environmental Health Sciences, Mailman School of Public Health, Columbia University, New York, United States of America

^2^ Q Partnership, Harare, Zimbabwe

^3^ Faculty of Public Health and Policy, Department of Global Health and Development, London School of Hygiene and Tropical Medicine, London, United Kingdom

^4^ Department of Art Design and Technology Education, University of Zimbabwe, Harare, Zimbabwe

^5^ Child Rights Research Centre, Africa University, Harare, Zimbabwe

**Corresponding author:**

[it2208@caa.columbia.edu](mailto:it2208@caa.columbia.edu)
