# Supplementary material for: National COVID-19 lockdown and trends in help-seeking for violence against children in Zimbabwe: an interrupted time-series analysis
Source: BMC Public Health. 2022 Nov 18;22:2116. doi: 10.1186/s12889-022-14425-w (PMC9673211; doi:10.1186/s12889-022-14425-w)
Supplement: Supplementary file 4 — Additional file 4. Modelling of violence call patterns [file 12889_2022_14425_MOESM4_ESM.docx]

**Additional file 4.** **Modelling of violence call patterns**

1. Visualisation of potential residual autocorrelation after model fitting

(A) *Lockdown model*


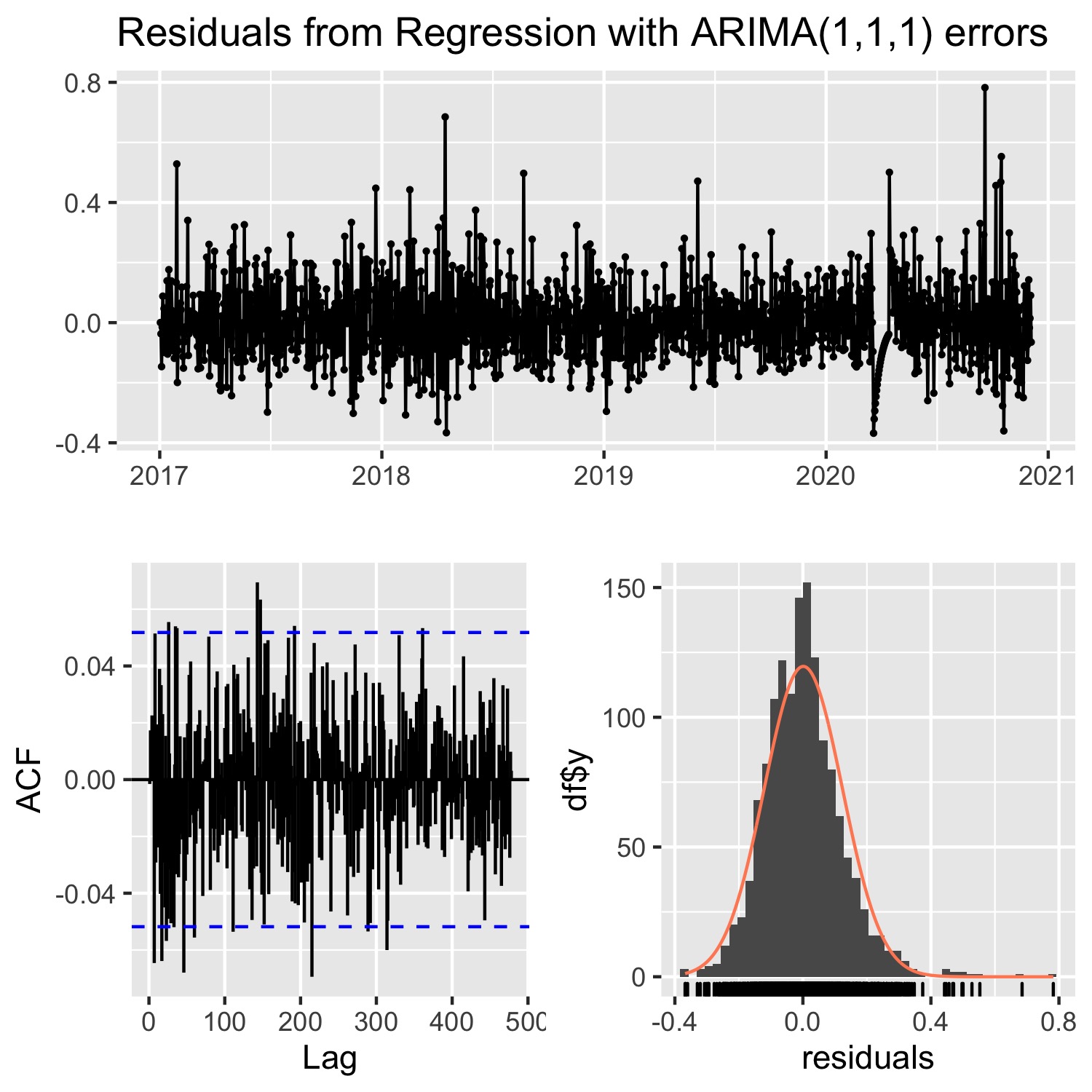


(B) *90-day model*


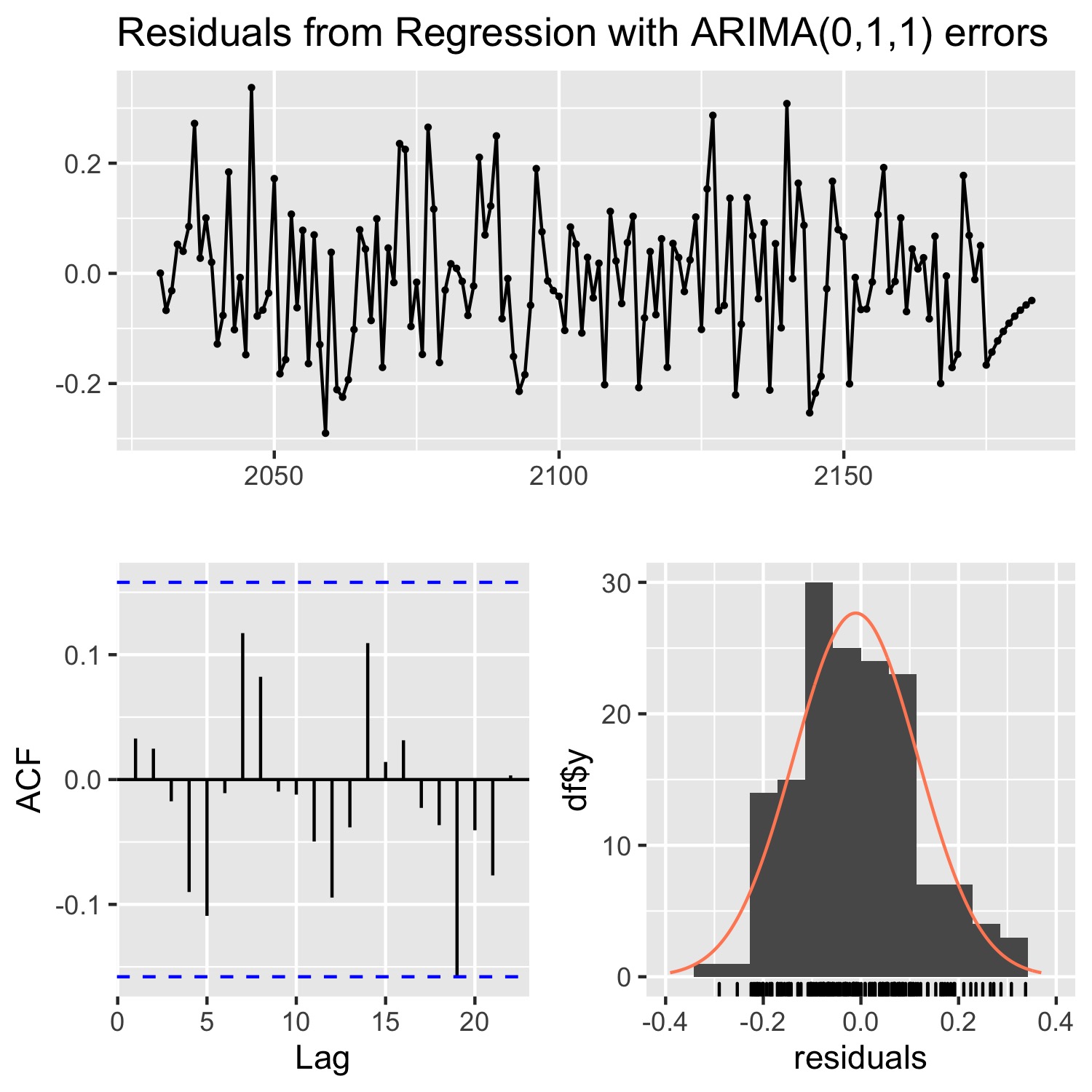


(C) *Monthly average model*


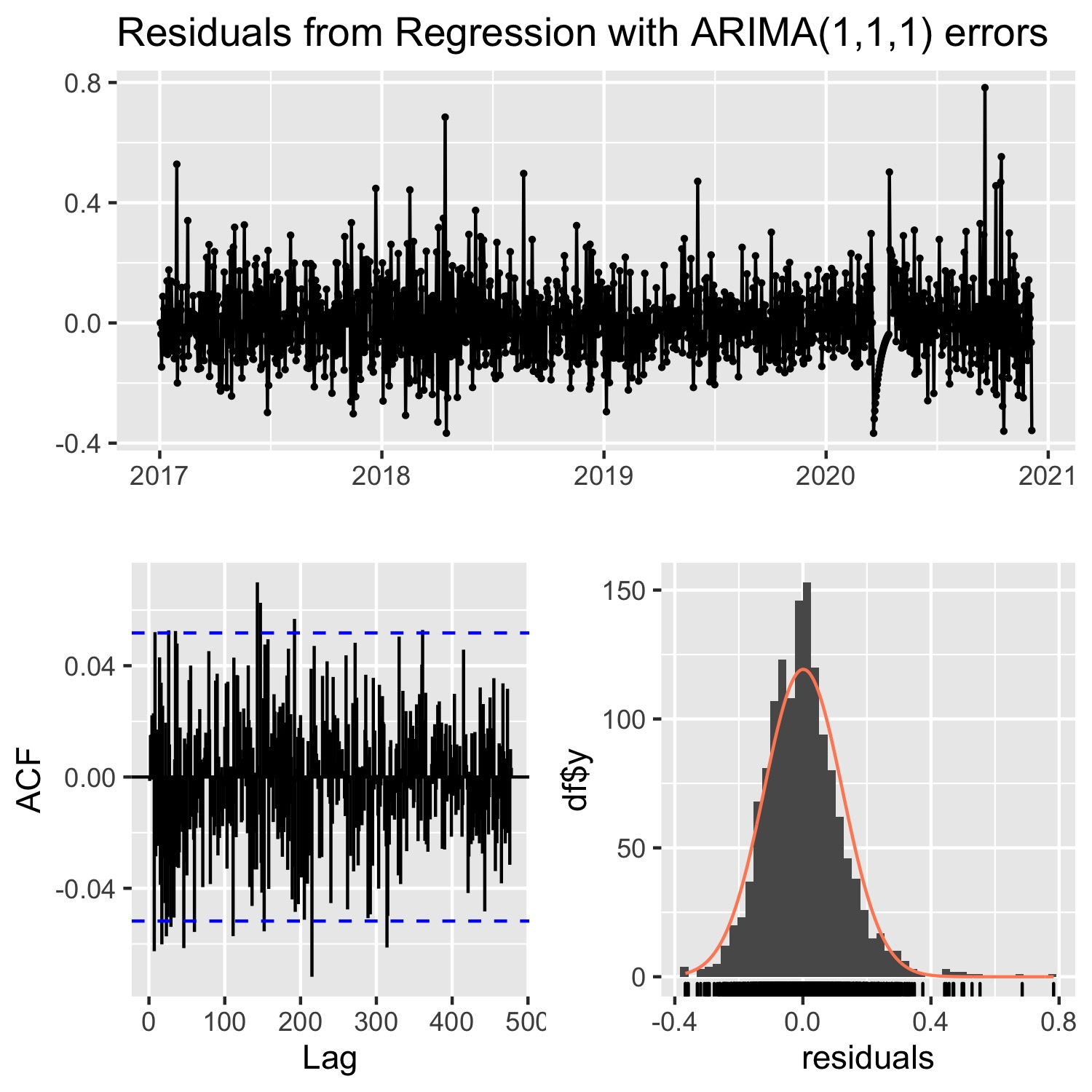


^a^ Lag indicates specific time points. ACF Lag = the correlation between the proportion of violence calls at each time point and proportions for previous days. Blue dotted line = a significant autocorrelation at *P*-value < 0.5. The symmetry and centring of the histogram around 0.00 indicates normality in the distribution of residuals.

**Article title:**

National COVID-19 lockdown and trends in help-seeking for violence against children in Zimbabwe: an interrupted time-series analysis

**Journal name:**

BMC Public Health

**Author names:**

Ilan Cerna-Turoff^1,3*^, Robert Nyakuwa^2^, Ellen Turner^3^, Charles Muchemwa Nherera^4^, Tendai Nhenga-Chakarisa^5^, Karen Devries^3^

**Affiliations:**

^1^ Department of Environmental Health Sciences, Mailman School of Public Health, Columbia University, New York, United States of America

^2^ Q Partnership, Harare, Zimbabwe

^3^ Faculty of Public Health and Policy, Department of Global Health and Development, London School of Hygiene and Tropical Medicine, London, United Kingdom

^4^ Department of Art Design and Technology Education, University of Zimbabwe, Harare, Zimbabwe

^5^ Child Rights Research Centre, Africa University, Harare, Zimbabwe

**Corresponding author:**

[it2208@caa.columbia.edu](mailto:it2208@caa.columbia.edu)
